# Genome-wide association analysis of four yield-related traits using a maize (*Zea mays L*.) F1 population

**DOI:** 10.1371/journal.pone.0305357

**Published:** 2024-06-25

**Authors:** Yong Zhang, Ziru Zeng, Feifei Tuo, Jin Yue, Zhu Wang, Weiming Jiang, Xue Chen, Xianya Wei, Qunkai Niu

**Affiliations:** School of Agronomy and Horticulture, Chengdu Agricultural College, Chengdu, Sichuan, China; KGUT: Graduate University of Advanced Technology, ISLAMIC REPUBLIC OF IRAN

## Abstract

Increasing the yield of maize F1 hybrid is one of the most important target for breeders. However, as a result of the genetic complexity and extremely low heritability, it is very difficult to directly dissect the genetic basis and molecular mechanisms of yield, and reports on genetic analysis of F1 hybrid yield are rare. Taking F1 hybrid as the research object and dividing the yield into different affect factors, this approach may be the best strategy for clarifying the genetic mechanism of yield. Therefore, in this study, a maize F1 population consisting of 300 hybrids with 17,652 single nucleotide polymorphisms (SNPs) markers was used for genome-wide association study (GWAS) to filtrate candidate genes associated with the four yield-related traits, i.e., kernel row number (KRN), kernel number per row (KNPR), ear tip-barrenness (ETB), and hundred kernel weight (HKW). Combined with the results of previous studies and functional annotation information of candidate genes, a total of six candidate genes were identified as being associated with the four traits, which were involved in plant growth and development, protein synthesis response, phytohormone biosynthesis and signal transduction. Our results improve the understanding of the genetic basis of the four yield-related traits and may be provide a new strategy for the genetic basis of maize yield.

## Introduction

Maize (*Zea mays L*.) is one of three major global grain crops and is also a main food source for humans and domestic animals. Therefore, increasing maize grain yield is a long-term and essential target, especially considering the concerns related to food safety and decreased yields in cultivated areas. During the past several decades, maize grain yield has increased approximately eightfold [1]. The significant increase in maize grain yield is not the result of advanced technology and management, but rather due to the hybrid breeding and utilization of F1 populations in agricultural production [1, 2]. In maize, yield has low heritability and is an extremely complex quantitative trait controlled by multiple genes with low effects; it is also affected by many factors [3, 4]. The genetic complexity and extremely low heritability make it almost impossible to directly dissect the genetic basis and molecular mechanisms of yield. This impedes our understanding of this trait. In maize, kernel row number (KRN), kernel number per row (KNPR), ear tip-barrenness (ETB), and hundred kernel weight (HKW) are four yield-related traits and are also important components of high-yield. Compared to the yield trait itself, the four agronomic traits are easy to study due to their high heritability. The yield is divided into different affect factors for genetic analysis, and this could be the best strategy for clarifying the genetic mechanisms of yield. Therefore, dissecting the genetic basis of the four agronomic traits instead of yield itself will provide a better understanding of the genetic basis of yield and might facilitate improvements in the utilization of this trait.

Quantitative trait loci (QTL) mapping is an effective tool for elucidating the genetic basis of complex quantitative traits. Currently, many QTLs related to the four yield-related traits in maize have been identified. For instance, An et al. identified a major QTL for KRN which was mapped to the region of 11.8–13.7 Mb on chromosome 5 by the F2 segregation population constructed by N351 and IL3455 [5]. Bommert et al. identified one major QTL for KRN on chromosome 4 using 250 recombinant inbred line (RIL) populations from B73 and Mo17 [6]. Lu et al. identified 13 QTLs for KRN in an F2:3 population consisting of 397 individuals in seven environments [7]. Tian et al. identified 13 QTLs for KRN using F2:3 and F2:4 populations from two maize inbred lines in different environments [8]. Sabadin et al. identified 5 QTLs for KNPR using an F2:3 tropical maize population consisting of 400 individuals in five environments [9]. Yang et al. identified 3 major QTLs for KNPR using a set of doubled haploid (DH) lines derived from the elite maize hybrid Zhengdan 958 [10]. Jia et al. identified a major QTL for KNPR on chromosome 6 in an F2 population [11]. Nie et al. Identified 5 QTLs for ETB an F2 population consisting of 150 individuals in two environments [12]. Ding et al. identified 3 QTLs for ETB using an F2:3 population consisting of 225 individuals in two environments [13]. Tang et al. identified 5 QTLs for HKW using an "immortalized F2" (IF2) maize population that included 441 individuals [14]. Raihan et al. identified 19 QTLs for HKW using a maize recombinant inbred line (RIL) population derived from a cross between two diverse parents Zheng58 and SK [15]. Although the four yield-related traits have been extensively studied in maize using different maize populations, the genetic basis remains unclear in the F1 population, a population that is widely used in agricultural production.

A genome-wide association study (GWAS) based on linkage disequilibrium (LD) is also an effective tool for assessing the genetic basis of complex quantitative traits. Studies on the yield-related traits in an F1 population by GWAS were rare. Therefore, in the present study, we used the F1 population as the association population to identify candidate genes related to the four agronomic traits in multiple environments by GWAS and to dissect the genetic basis of these traits. An F1 population that consisted of 300 maize single crosses, was constructed through anomalous diallel crosses using 99 maize inbred lines genotyped by genotyping-by-sequencing (GBS) technology [16]. The four agronomic traits, namely KRN, KNPR, ETB, and HKW, were measured in four environments for one year. The present study aimed to improve the understanding of the genetic basis of the four agronomic traits and provide new insights into the genetic basis of yield in maize.

## Materials and methods

### Materials and field trials

In this study, the F1 population for the GWAS consisted of 300 maize single crosses that were derived from 99 maize inbred lines by an anomalous diallel cross method. The hybridization pattern of 300 hybrids were shown in S1 Table. The 300 maize hybrids were planted in a completely randomized block design with two replicates per environment. Each hybrid was planted in a single row. Each row contained 10 plants, and 2 plants were grown in each pit. Each row was 2 m in length and 0.80 m from the next row, and the planting density was approximately 60,000 individuals/ha. The four yield-related traits including KRN, KNPR, ETB, and HKW, were measured in the following locations of China in 2017, including Mianyang (E104°44’, N31°28’), Luding (E102°14’, N29°54’), Yibin (E104°37’, N28°45’), and Ya’an (E103°0’, N29°59’). These traits were evaluated in each hybrid by randomly selecting five maize ears that were developing normally. The trait phenotype value for each hybrid was the average of the measured ears. The field trial was conducted using conventional field management.

### Analysis of phenotypic data

Analysis of variance (ANOVA) of the phenotypic data for the four traits was performed with IBM SPSS Statistics version 20.0 software. The R package “lme4” was used for computing the best linear unbiased prediction (BLUP) values in the four different locations [17]. Correlation analysis and descriptive statistical analysis of the BLUP phenotype data for the four traits were also performed with IBM SPSS Statistics version 20.0 software. The broad-sense heritability (H^2^) was calculated as described by Knapp [18] as H^2^ = V_G_/(V_G_ + V_GE_/*n* + V_residual_/*rn*), where V_G_, V_GE_, and V_residual_ are estimates of the genotypic variance, the interaction variance of genotype × environment, and the error variance, respectively, and *n* and *r* are the number of environments and the number of replications per environment, respectively.

### Analysis of genotypic data

The genotypes of 99 inbred lines were sequenced by genotyping-by-sequencing (GBS) technology [16], and a total of 559,678 SNPs markers were obtained. In 99 inbred lines, SNPs markers that were heterozygous or missing were deleted; only SNPs markers that were homozygous were retained. The genotypes of the hybrids, which were inferred according to the genotype of their parents, were numerically coded and used in GWAS (2, 1, and 0 for AA, Aa, and aa, respectively) [19];. Finally, the genotypes of 300 hybrids containing 17,652 SNPs markers were used for population structure analysis, kinship coefficients analysis, LD calculation, and GWAS. The analysis results of LD, population structure (*Q* matrix), and kinship (*K* matrix) of the 300 hybrids were presented in our published article (S1–S3 Figs) [20]. In addition, the average LD attenuation distance among 10 chromosomes was 200 kb (R^2^ = 0.2) [20]. The analysis method of genotypic data was described in detail in our published article (https://www.mdpi.com/2223-7747/8/10/432) [20].

### Genome-wide association study

GWAS analysis for the four yield-related traits was performed using the FarmCPU model, a fixed and random effect model in which population structure (*Q* matrix) is a fixed effect and kinship (*K* matrix) is a random effect [21]. SNP markers and the traits were considered to be significantly associated only if the *P* value was lower than the threshold *P*_threshold_ = -log10 (0.05 / *N*), where *N* is the total number of SNP markers [22]. Therefore, the threshold level for trait-marker significant associations was set as 5.58 in this study. The whole genome sequence of the maize B73 (B73_ RefGen_v4, https://www.maizegdb.org/) [23] was used as a reference genome for identifying candidate genes [24]. Based on the physical position on the chromosome of the SNP markers that was significantly associated with the target trait, the maizeGDB database (B73_RefGen_v4) was used for identifying candidate genes related to the target trait. Herein, we searched the candidate genes based on the region of the average LD decay among 10 chromosomes.

## Results

### Phenotypic analysis and heritability

**Fig 1:**
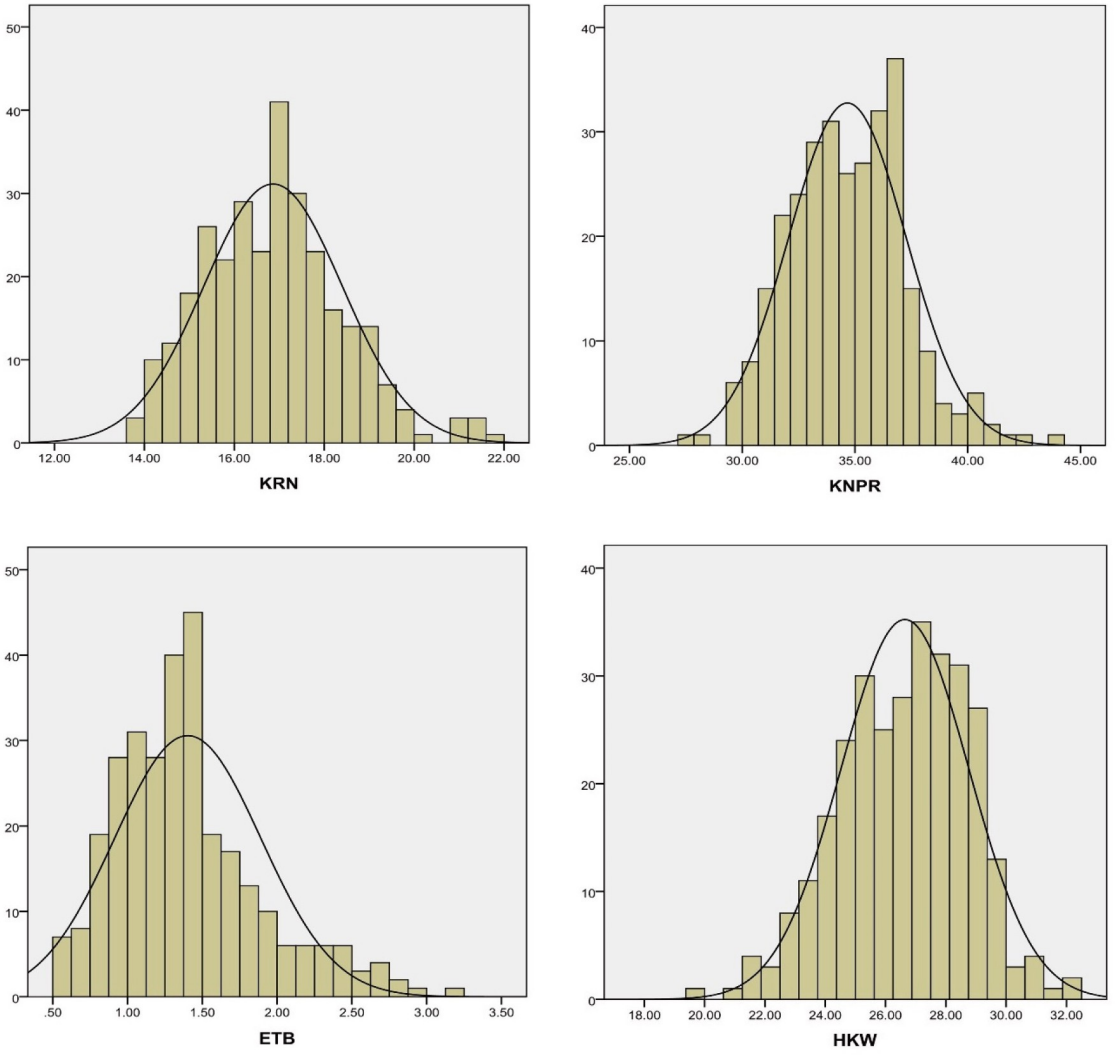
The BLUP phenotype distribution of the four traits. KRN: kernel row number, KNPR: kernel number per row, ETB: ear tip-barrenness, HKW: hundred kernel weight.

The descriptive statistical analysis and correlation analysis of the BLUP phenotype data of the four traits are shown in Table 1. The skewness and kurtosis of the four traits were all between -1 and 1, indicating that these traits all conformed to the normal distribution (Fig 1). Moreover, the results indicated that the four traits were quantitative traits controlled by multi-genes with low effects. ANOVA of the phenotypic data showed that the four traits were collectively affected by genotype, environment, and genotype × environment interaction (Table 2). The H^2^ of KRN was 88%, indicating that KRN was mainly controlled by genetics. Correlation analysis of the four traits suggested that these traits should be coordinately developed when considering the improvement in yield (Table 3). The above results indicated that the measured phenotype data are reliable for dissecting the genetic basis of the four traits.

**Table 1:** Descriptive statistical analysis of the BLUP phenotype and the broad-sense heritability of the four traits.

Trait	Mean	Maximum	Minimum	SD	Skewness	Kurtosis	CV(%)	H^2^(%)
KRN	16.86	21.96	13.74	1.54	0.44	0.22	9.13	88.00
KNPR	34.66	43.96	27.34	2.61	0.25	0.30	7.53	70.52
ETB	1.40	3.24	0.51	0.49	0.93	0.91	35.00	66.63
HKW	26.64	32.50	19.57	2.12	-0.24	-0.08	7.96	67.35

KRN: kernel row number, KNPR: kernel number per row, ETB: ear tip-barrenness, HKW: hundred kernel weight, SD: standard deviation, CV: coefficient of variation, H^2^: the broad-sense heritability

**Table 2:** Analysis of variance for the four traits.

Trait	Source	SS	DF	MS	*F* value	Sig
KRN	G	6279.14	299	21.00	34.68	<0.01 **
	E	982.783	3	327.59	540.98	<0.01 **
	G×E	1099.15	893	1.23	2.03	<0.01 **
	Error	703.06	1161	0.61		
KNPR	G	28,047.99	299	93.81	17.89	<0.01 **
	E	4288.07	3	1429.36	272.55	<0.01 **
	G×E	20,385.84	893	22.83	4.35	<0.01 **
	Error	6088.70	1161	5.24		
ETB	G	1005.34	299	3.36	13.87	<0.01 **
	E	379.51	3	126.51	521.84	<0.01 **
	G×E	761.71	893	0.85	3.52	<0.01 **
	Error	281.45	1161	0.24		
HKW	G	19,850.25	299	66.39	15.70	<0.01 **
	E	8747.83	3	2915.94	689.67	<0.01 **
	G×E	16,449.92	890	18.48	4.37	<0.01 **
	Error	4866.46	1151	4.23		

KRN: kernel row number, KNPR: kernel number per row, ETB: ear tip-barrenness, HKW: hundred kernel weight, SS: sum of squares, DF: degree of freedom, MS: mean square, Sig: significance level, G: genotype, E: environment, G × E: genotype × environment

** indicates significance at a level of 0.01.

There are more missing values in HKW, resulting in differences in DF compared to the other three traits.

**Table 3:** Correlation analysis for the four traits.

Trait	KRN	KNPR	ETB	HKW
KRN	1	-0.218 **	0.018	-0.224**
KNPR		1	-0.226 **	-0.002
ETB			1	-0.222**
HKW				1

KRN: kernel row number, KNPR: kernel number per row, ETB: ear tip-barrenness, HKW: hundred kernel weight

** indicates significance at a level of 0.01.

#### GWAS analysis

**Fig 2:**
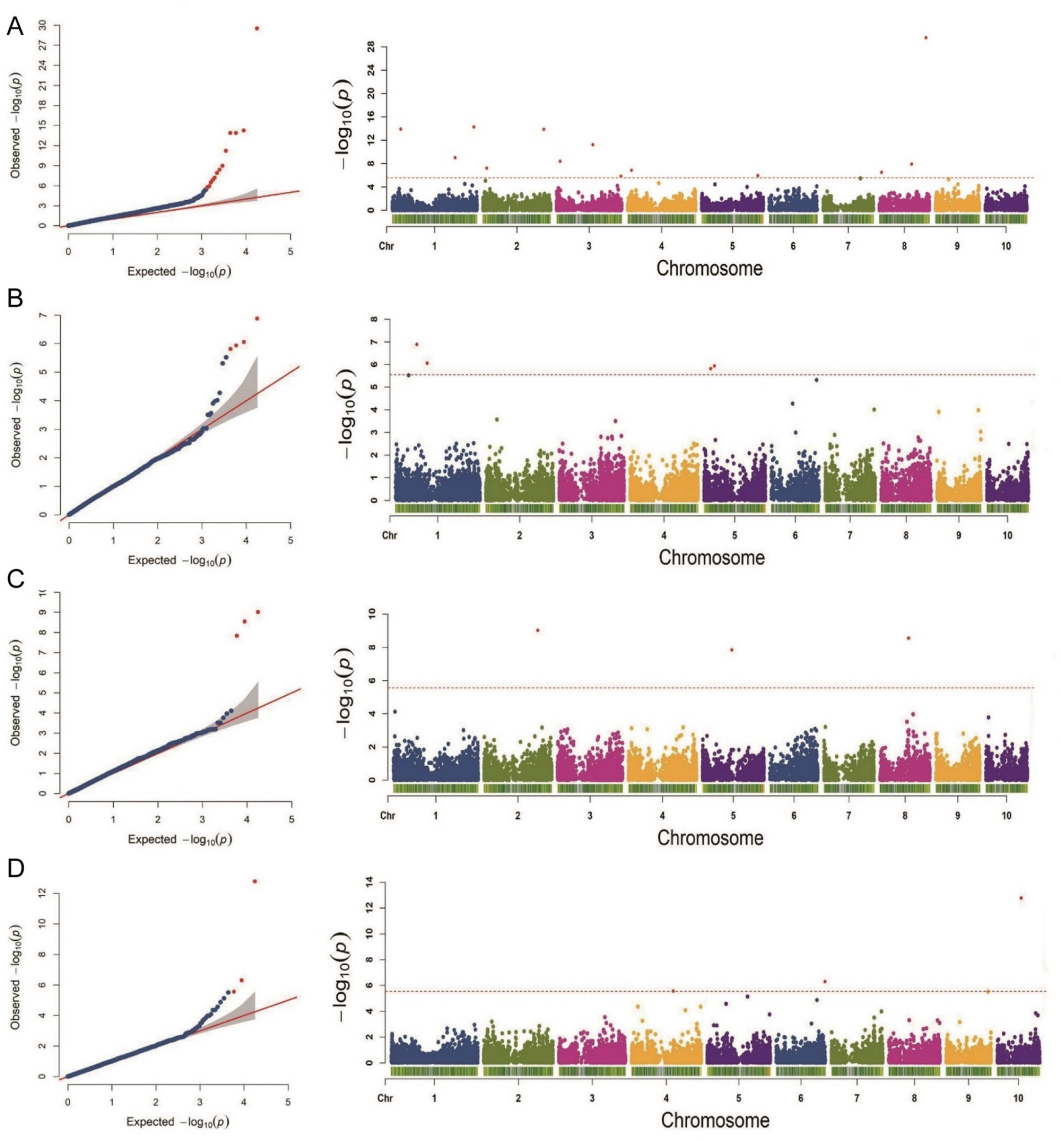
Quantile-quantile plot and Manhattan plot of the four traits. (A) KRN. (B) KNPR. (C) ETB. (D) HKW. KRN: kernel row number, KNPR: kernel number per row, ETB: ear tip-barrenness, HKW: hundred kernel weight.

The BLUP phenotype data of the four traits and the genotype data of the 300 hybrids were used for GWAS by the FarmCPU model. In this study, the GWAS used 17,652 SNPs markers to elucidate the genetic basis of the four traits. We used the *Q* + *K* model to identify candidate genes to reduce the number of false positives. Therefore, the *Q* matrix (population structure) and the *K* matrix (kinship) were fitted to the FarmCPU model to control the number of the false positives in this study. Quantile-quantile (Q-Q) plots showed that the *Q* matrix (population structure) and the *K* matrix (kinship) were well controlled for GWAS in the FarmCPU model (Fig 2). A total of 23 SNP markers significantly associated with the four traits were identified by the FarmCPU model (Fig 2). Thirteen SNP markers that were significantly associated with KRN were located on chromosomes 1, 2, 3, 4, 5, and 8 (Table 4). Four SNP markers that were significantly associated with KNPR were located on chromosomes 1 and 5 (Table 4). Three SNP markers that were significantly associated with ETB were located on chromosomes 2, 5, and 8 (Table 4). Three SNP markers that were significantly associated with HKW were located on chromosomes 4, 6, and 10 (Table 4).

**Table 4:** Summary of the significant markers for the four traits.

Trait	Chr	SNP ID	Allele	SNP physical position	*P* value
KRN	1	SNP-11455	A/G	30,518,448	1.34 × 10^−14^
	1	SNP-59129	A/T	227,157,748	1.03 × 10^−9^
	1	SNP-78440	C/T	294,619,532	5.56 × 10^−15^
	2	SNP-90543	G/A	13,575,608	6.36 × 10^−8^
	2	SNP-141680	C/T	219,963,025	1.36 × 10^−14^
	3	SNP-155371	A/T	13,020,058	4.13 × 10^−9^
	3	SNP-180302	C/G	131,552,208	6.13 × 10^−12^
	3	SNP-211002	C/T	233,051,305	1.60 × 10^−6^
	4	SNP-217197	A/G	14,333,508	1.46 × 10^−7^
	5	SNP-326673	C/G	203,140,673	1.25 × 10^−6^
	8	SNP-433173	C/T	8,620,128	3.49 × 10^−7^
	8	SNP-458200	G/A	116,653,499	1.26 × 10^−8^
	8	SNP-473464	A/G	168,712,402	2.92 × 10^−30^
KNPR	1	SNP-24335	A/G	77,399,197	1.31 × 10^−7^
	1	SNP-32093	A/G	114,513,587	8.82 × 10^−7^
	5	SNP-285801	C/T	24,818,951	1.54 × 10^−6^
	5	SNP-289119	T/C	38,174,230	1.17 × 10^−6^
ETB	2	SNP-133200	A/G	194,431,509	9.59 × 10^−10^
	5	SNP-304599	G/A	106,333,721	1.45 × 10^−8^
	8	SNP-455169	G/C	103,255,196	2.82 × 10^−9^
HKW	4	SNP-246400	C/T	147,818,292	2.78 × 10^−6^
	6	SNP-381677	A/T	172,323,256	5.00 × 10^−7^
	10	SNP-538500	G/T	85,285,984	1.65 × 10^−13^

KRN: kernel row number, KNPR: kernel number per row, ETB: ear tip-barrenness, HKW: hundred kernel weight

### Candidate genes analysis

Based on the average LD attenuation distance among the 10 chromosomes, we searched the candidate genes in the range of 200 kb upstream and 200 kb downstream of the significant SNP markers. Combining the functional annotations of the candidate genes with the results of previous studies, we identified six candidate genes that are likely to be potential genes associated with the four traits, including three candidate genes for KRN, one candidate gene for KNPR, one candidate gene for ETB and one candidate gene for HKW. The details of the candidate genes and the functional annotations are shown in Table 5.

**Table 5:** The functional annotations of the candidate genes identified in this study.

Trait	Chr	SNP ID	Gen ID	Encoding
KRN	1	SNP-11455	*Zm00001d028317*	Leucine-rich repeat receptor-like serine/threonine-protein kinase BAM3
	5	SNP-326673	*Zm00001d017649*	S-ribonuclease binding protein SBP1
	2	SNP-90543	*Zm00001d002468*	Auxin responsive protein
KNPR	5	SNP-285801	*Zm00001d013919*	Tetratricopeptide repeat (TPR)-like superfamily protein
ETB	2	SNP-133200	*Zm00001d005978*	RING-H2 finger protein
HKW	6	SNP-381677	*Zm00001d039188*	Putative leucine-rich repeat receptor-like protein kinase family protein

KRN: kernel row number, KNPR: kernel number per row, ETB: ear tip-barrenness, HKW: hundred kernel weight

## Discussion

Maize yield is the most important breeding trait. However, yield is an extremely complex quantitative trait, which is controlled by multiple genes with low effects and has a lower H^2^ than other quantitative traits. It is thus extremely difficult to directly dissect the genetic basis of yield. KRN, KNPR, ETB, and HKW are four yield-related traits that have a higher heritability (H^2^) than the yield trait. Therefore, it is feasible to improve the understanding of the genetic mechanism of yield by dissecting the genetic basis of the four traits. Among these four traits, the H^2^ of KRN was highest, nearly reaching 90%. This finding was consistent with that of previous studies [6–8, 14], indicating that KRN is mainly controlled by genetic factors and is suitable for early generation selection. Moreover, the phenotypic data of the four traits all conformed to a normal distribution. Therefore, the four traits are suitable for statistical analysis. By exploring the genetic architecture of these four traits, the present study provides new insights into the genetic basis of yield in maize.

Various types of maize populations have been constructed to evaluate the genetic basis of complex quantitative traits, including natural populations, bi-parental populations, multi-parental populations, and mating-design populations [25]. Different populations have different advantages and disadvantages in the genetic analysis of quantitative traits. Natural populations consisting of maize inbred lines have been widely used for the GWAS of complex quantitative traits. However, study results based on maize inbred lines cannot be directly used to reveal the genetic mechanisms in maize F1 hybrid. The maize F1 hybrids have been widely used in agricultural production and were also important for stable grain yield. Compared to the natural population, the F1 population has the following advantages. First, the F1 population demonstrates greater adaptability and stronger vitality to different environments as a result of heterosis. Thus, the F1 population can possibly be used in trials across multiple environments. Second, using the F1 population can reduce the cost of genotyping. Currently, genotyping remains very expensive, but using the F1 population can greatly reduce the cost of sequencing. As only the parental inbred lines need to be genotyped, the F1 genotype can be inferred according to the bi-parental genotype [19]. This would greatly reduce the cost of sequencing [20]. Third, heterosis is most obvious in the F1 population, thus the F1 population can be used to directly study heterosis.

Currently, GWAS based on LD has become an efficient method for identifying the candidate genes related to the quantitative traits controlled by a number of small-effect QTLs. Moreover, the existence of various types of mutants allows for the possible dissection the genetic basis of extremely complex quantitative traits. In this study, we identified six candidate genes that are most likely associated with the four traits of interest according to previous studies. Taguchi-Shiobara et al. cloned the *fea2* gene, which encodes a leucine-rich repeat receptor-like protein and controls maize KRN by regulating the size of the inflorescence meristem [26]. Chuck et al. identified that the SBP-box transcription factors unbranched2 and unbranched3 cause an increase in ear row number [27]. Auxin plays a key role in the development of leafhopper meristem [28]. In this study, the candidate gene *Zm00001d028317* encodes a leucine-rich repeat receptor-like serine/threonine-protein kinase BAM3 that is related to a leucine-rich repeat receptor-like protein; the candidate gene *Zm00001d017649* encodes S-ribonuclease binding protein SBP1, which is related to the SBP-box protein; and the candidate gene *Zm00001d002468* encodes an auxin responsive protein that is related to auxin. Therefore, we proposed that the candidate genes *Zm00001d028317*, *Zm00001d017649* and *Zm00001d00246*8 were the most likely potential genes involved in the development of KRN. In tomato, Arabidopsis and soybean, tetratricopeptide repeat (TPR)-like superfamily proteins have been demonstrated to interact with ethylene receptors, play a crucial role in ethylene signaling, and participate in the interaction between ethylene and auxin [29–31]. Moreover, Hays et al. reported that ethylene induced kernel abortion in wheat [32]. In this study, the candidate gene *Zm00001d013919* encodes a TPR-like superfamily protein. Therefore, we proposed that the candidate gene *Zm00001d013919* was the most likely potential gene involved in the development of KNPR. In rice, the gene *GW2*, which encodes a RING-type protein with E3 ubiquitin ligase activity, controls the development of rice grains. Song et al. discovered that the loss of *GW2* function accelerated the grain milk filling rate [33]. In this study, the candidate gene *Zm00001d005978* encodes a RING-H2 finger protein, which is related to a RING-type protein. Therefore, we proposed that the candidate gene *Zm00001d005978* is the most likely potential gene involved in the development of ETB. In maize, Chen et al. identified that *GRMZM2G039934*, which encodes a putative leucine-rich repeat receptor-like protein kinase family protein, is the most likely candidate gene for *qGW4*.*05*, a major QTL for kernel weight and size [34]. In this study, the candidate gene *Zm00001d039188* also encodes a putative leucine-rich repeat receptor-like protein kinase family protein. Therefore, we suggested that the candidate gene *Zm00001d039188* is most likely to be involved in the development of HKW.

In summary, a total of six candidate genes were identified as associated with the four traits. Three candidate genes for KRN were identified on chromosomes 1, 2, and 5. One candidate gene for KNPR was identified on chromosome 5; one candidate gene for ETB was identified on chromosome 2; and one candidate gene for HKW was identified on chromosome 6.

## Conclusion

In our study, the F1 population consisting of 300 hybrids was used for GWAS using the FarmCPU model, and a total of 23 significant SNPs markers associated with the four traits were detected. According to the physical location of significant SNPs markers, six candidate genes were identified, and these candidate genes will be the subject of next study.

## Supporting information

S1 Table The hybridization pattern of 300 hybrids that were derived from 99 maize inbred lines. CK = check or control.
(DOCX)

S1 Fig LD decay rate per chromosome based on mean R^2^ per 100 kb region. (TIF)

S2 Fig Analysis of the population structure of the 300 hybrids estimated from 17,652 SNPs. (A) ΔK value related to different K; (B) Population structure of the 300 hybrids from K = 4.
(TIF)

S3 Fig The distributions of kinship between 300 hybrids. (TIF)

## Abbreviations

BLUP: best linear unbiased prediction
ETB: ear tip-barrenness
GBS: genotyping-by-sequencing
GWAS: genome-wide association analysis
HKW: hundred kernel weight
KNPR: kernel number per row
KRN: kernel row number
QTLs: quantitative trait loci
SNPs: single nucleotide polymorphisms
