# Supplementary material for: Genome-wide association analysis of four yield-related traits using a maize (Zea mays L.) F1 population
Source: PLoS One. 2024 Jun 25;19(6):e0305357. doi: 10.1371/journal.pone.0305357 (PMC11198826; doi:10.1371/journal.pone.0305357)
Supplement: S1 Table — CK = check or control. (DOCX) [file pone.0305357.s001.docx]

S1 Table. The hybridization pattern of 300 hybrids that were derived from 99 maize inbred lines. CK = check or control.

| Hybrids ID | Hybridization Pattern | Hybrids ID | Hybridization Pattern | Hybrids ID | Hybridization Pattern |
| --- | --- | --- | --- | --- | --- |
| H1(CK)  H2  H3  H4  H5  H6  H7  H8  H9  H10  H11  H12  H13  H14  H15  H16  H17  H18  H19  H20  H21  H22  H23  H24  H25  H26  H27  H28  H29  H30  H31  H32  H33  H34  H35  H36  H37  H38  H39  H40  H41  H42  H43  H44  H45  H46  H47  H48  H49  H50  H51  H52  H53  H54  H55  H56  H57  H58  H59  H60  H61  H62  H63  H64  H65  H66  H67  H68  H69  H70  H71  H72  H73  H74  H75  H76  H77  H78  H79  H80  H81  H82  H83  H84  H85  H86  H87  H88  H89  H90  H91  H92  H93  H94  H95  H96  H97  H98  H99  H100 | L41×L93  L20×L32  L69×L27  L95×L68  L95×L43  L95×L55  L1×L28  L1×L29  L95×L42  L96×L93  L96×L47  L95×L47  L41×L39  L29×L95  L92×L58  L92×L72  L92×L95  L92×L5  L93×L95  L39×L93  L48×L21  L93×L18  L92×L55  L41×L31  L41×L32  L69×L95  L41×L33  L95×L34  L41×L34  L41×L35  L68×L36  L41×L37  L41×L52  L41×L38  L69×L30  L92×L39  L41×L40  L96×L41  L2×L68  L2×L92  L96×L68  L2×L42  L96×L51  L95×L58  L95×L66  L13×L34  L13×L44  L13×L18  L13×L26  L21×L47  L21×L44  L21×L26  L3×L68  L3×L23  L1×L47  L65×L47  L72×L47  L72×L48  L72×L93  L72×L26  L41×L92  L41×L49  L41×L42  L41×L48  L41×L50  L64×L52  L64×L47  L41×L26  L9×L26  L41×L53  L12×L58  L41×L54  L4×L92  L4×L18  L17×L58  L17×L26  L41×L55  L6×L47  L6×L42  L6×L93  L8×L47  L18×L8  L10×L42  L22×L56  L71×L57  L92×L24  L92×L22  L92×L23  L65×L59  L11×L60  L19×L41  L48×L22  L93×L22  L93×L61  L22×L62  L93×L26  L69×L63  L12×L64  L7×L47  L2×L47 | H101  H102  H103  H104  H105  H106  H107  H108  H109  H110  H111  H112  H113  H114  H115  H116  H117  H118  H119  H120  H121  H122  H123  H124  H125  H126  H127  H128  H129  H130  H131  H132  H133  H134  H135  H136  H137  H138  H139  H140  H141  H142  H143  H144  H145  H146  H147  H148  H149  H150  H151  H152  H153  H154  H155  H156  H157  H158  H159  H160  H161  H162  H163  H164  H165  H166  H167  H168  H169  H170  H171  H172  H173  H174  H175  H176  H177  H178  H179  H180  H181  H182  H183  H184  H185  H186  H187  H188  H189  H190  H191  H192  H193  H194  H195  H196  H197  H198  H199  H200 | L2×L23  L2×L57  L80×L47  L73×L47  L20×L47  L20×L46  L93×L20  L26×L45  L26×L95  L26×L47  L26×L46  L26×L22  L26×L68  L26×L49  L26×L44  L26×L58  L13×L20  L14×L26  L95×L94  L95×L25  L67×L90  L67×L85  L67×L83  L67×L76  L67×L34  L67×L94  L67×L48  L65×L93  L65×L92  L65×L94  L65×L84  L78×L93  L78×L92  L78×L94  L78×L25  L78×L48  L78×L55  L78×L42  L78×L90  L78×L62  L78×L26  L78×L74  L78×L89  L78×L77  L78×L53  L96×L76  L96×L92  L96×L94  L96×L25  L96×L48  L96×L32  L96×L42  L96×L62  L96×L85  L96×L89  L96×L83  L96×L53  L96×L99  L96×L15  L16×L76  L16×L62  L70×L93  L70×L92  L70×L94  L70×L25  L70×L32  L70×L42  L70×L62  L70×L26  L70×L77  L70×L83  L12×L76  L12×L93  L12×L34  L12×L92  L12×L94  L12×L48  L12×L55  L12×L42  L12×L62  L12×L26  L12×L89  L12×L77  L12×L53  L97×L83  L98×L94  L98×L48  L98×L32  L98×L90  L98×L62  L98×L26  L98×L85  L98×L74  L98×L77  L98×L83  L41×L76  L41×L94  L41×L90  L41×L15  L9×L93 | H201  H202  H203  H204  H205  H206  H207  H208  H209  H210  H211  H212  H213  H214  H215  H216  H217  H218  H219  H220  H221  H222  H223  H224  H225  H226  H227  H228  H229  H230  H231  H232  H233  H234  H235  H236  H237  H238  H239  H240  H241  H242  H243  H244  H245  H246  H247  H248  H249  H250  H251  H252  H253  H254  H255  H256  H257  H258  H259  H260  H261  H262  H263  H264  H265  H266  H267  H268  H269  H270  H271  H272  H273  H274  H275  H276  H277  H278  H279  H280  H281  H282  H283  H284  H285  H286  H287  H288  H289  H290  H291  H292  H293  H294  H295  H296  H297  H298  H299  H300 | L9×L92  L9×L94  L9×L32  L9×L42  L9×L62  L9×L85  L9×L89  L9×L84  L87×L48  L88×L76  L88×L34  L88×L94  L88×L48  L88×L90  L88×L62  L88×L26  L88×L85  L88×L77  L88×L84  L88×L83  L86×L76  L86×L94  L86×L48  L86×L32  L86×L90  L86×L62  L86×L26  L86×L77  L86×L83  L76×L98  L93×L67  L93×L97  L93×L98  L93×L79  L93×L82  L93×L87  L93×L88  L93×L86  L34×L87  L92×L76  L92×L97  L92×L98  L92×L75  L92×L82  L92×L87  L94×L82  L25×L82  L25×L87  L48×L95  L32×L95  L32×L97  L32×L79  L55×L67  L55×L16  L55×L75  L55×L82  L55×L87  L42×L67  L42×L65  L42×L16  L42×L97  L42×L98  L42×L87  L85×L97  L74×L97  L74×L86  L89×L95  L89×L67  L89×L65  L89×L16  L89×L97  L89×L75  L89×L86  L77×L67  L77×L65  L77×L97  L93×L99  L48×L99  L91×L65  L53×L95  L53×L86  L96×L26  L95×L90  L70×L34  L41×L74  L78×L34  L25×L16  L16×L34  L87×L77  L25×L67  L12×L74  L55×L65  L89×L87  L89×L81  L92×L86  L97×L62  L96×L90  L16×L94  L71×L29  L94×L87 |
