# Supplementary figures and images for: Genome-wide association analysis of four yield-related traits using a maize (Zea mays L.) F1 population
Source: PLoS One. 2024 Jun 25;19(6):e0305357. doi: 10.1371/journal.pone.0305357 (PMC11198826; doi:10.1371/journal.pone.0305357)

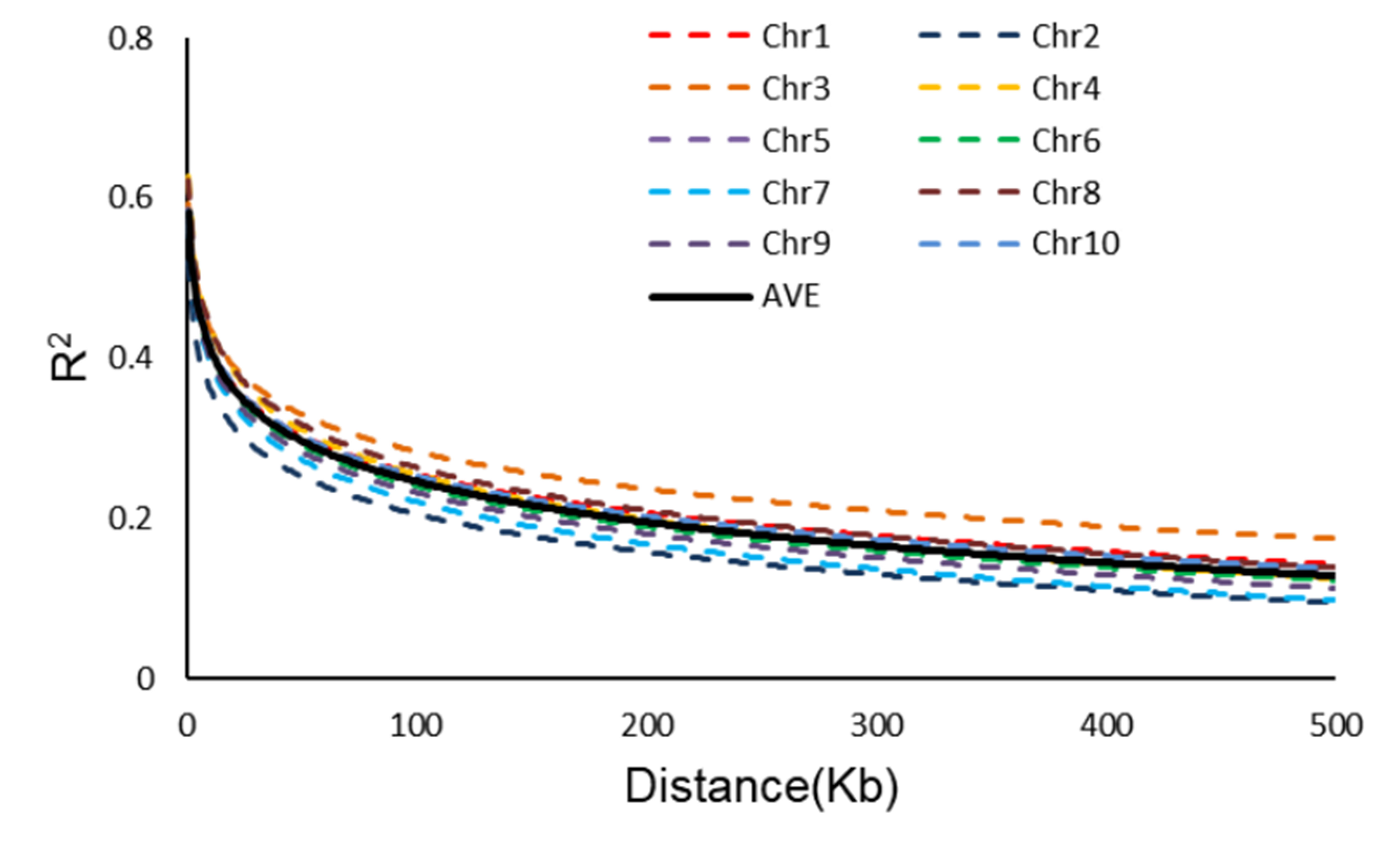

Supplement: S1 Fig — (TIF) [file pone.0305357.s002.tif]

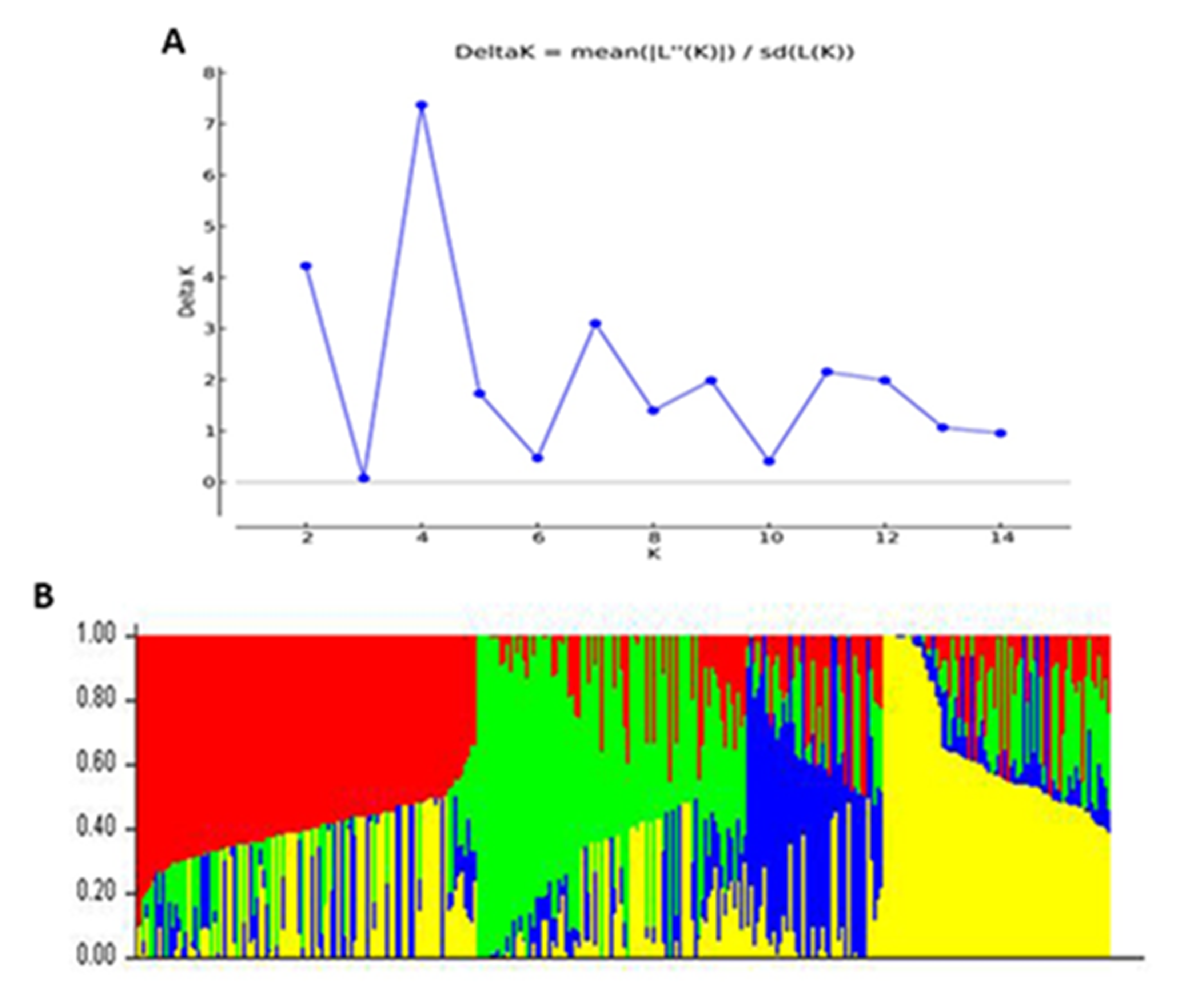

Supplement: S2 Fig — (A) ΔK value related to different K; (B) Population structure of the 300 hybrids from K = 4. (TIF) [file pone.0305357.s003.tif]
